# Supplementary figures and images for: Indoxyl sulfate, a gut microbiome-derived uremic toxin, is associated with psychic anxiety and its functional magnetic resonance imaging-based neurologic signature
Source: Sci Rep. 2021 Oct 25;11:21011. doi: 10.1038/s41598-021-99845-1 (PMC8546034; doi:10.1038/s41598-021-99845-1)

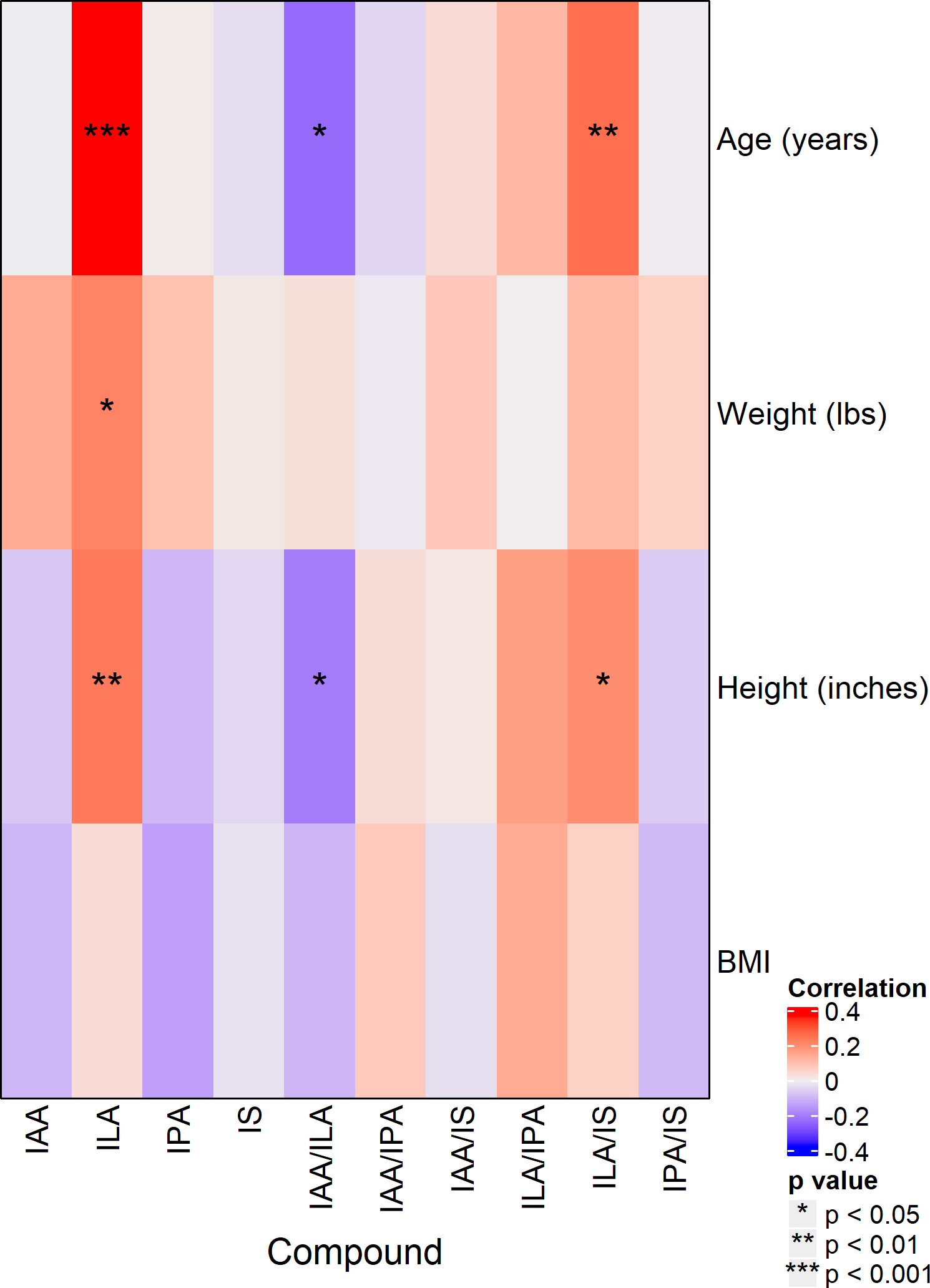

Supplement: Supplementary file 1 — Supplementary Figure 1. [file 41598_2021_99845_MOESM1_ESM.tiff]

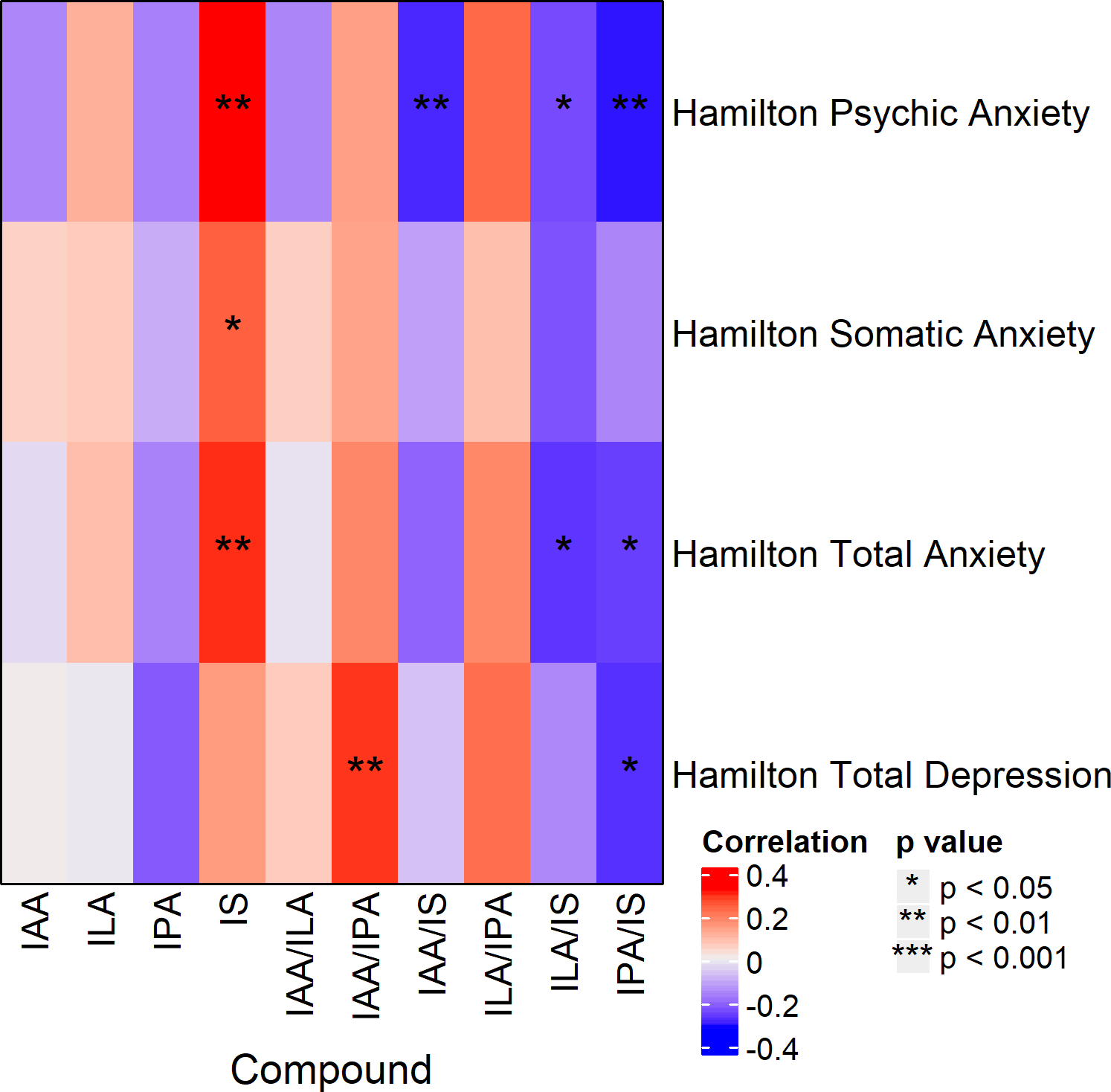

Supplement: Supplementary file 2 — Supplementary Figure 2. [file 41598_2021_99845_MOESM2_ESM.tiff]

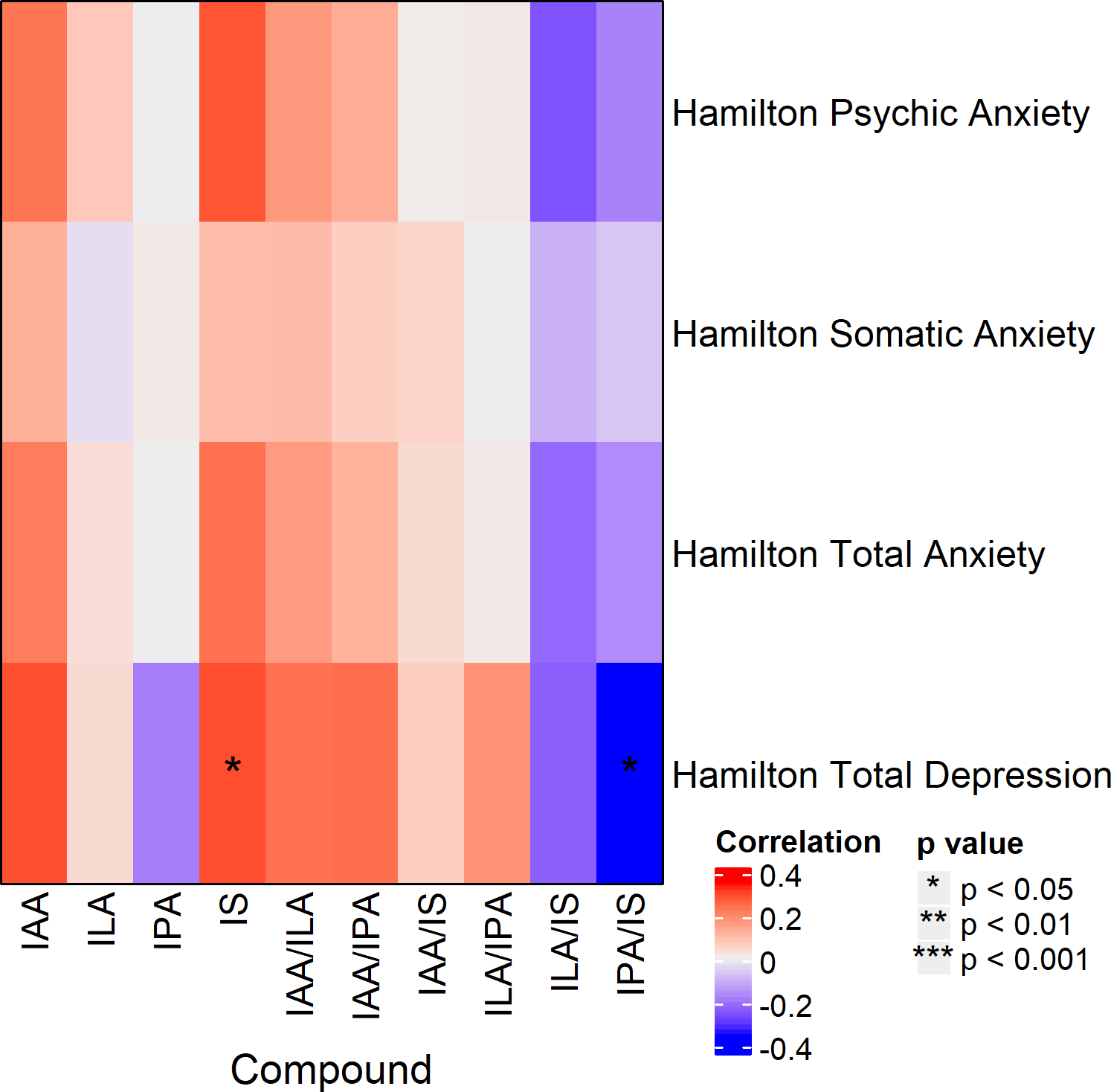

Supplement: Supplementary file 3 — Supplementary Figure 3. [file 41598_2021_99845_MOESM3_ESM.tiff]

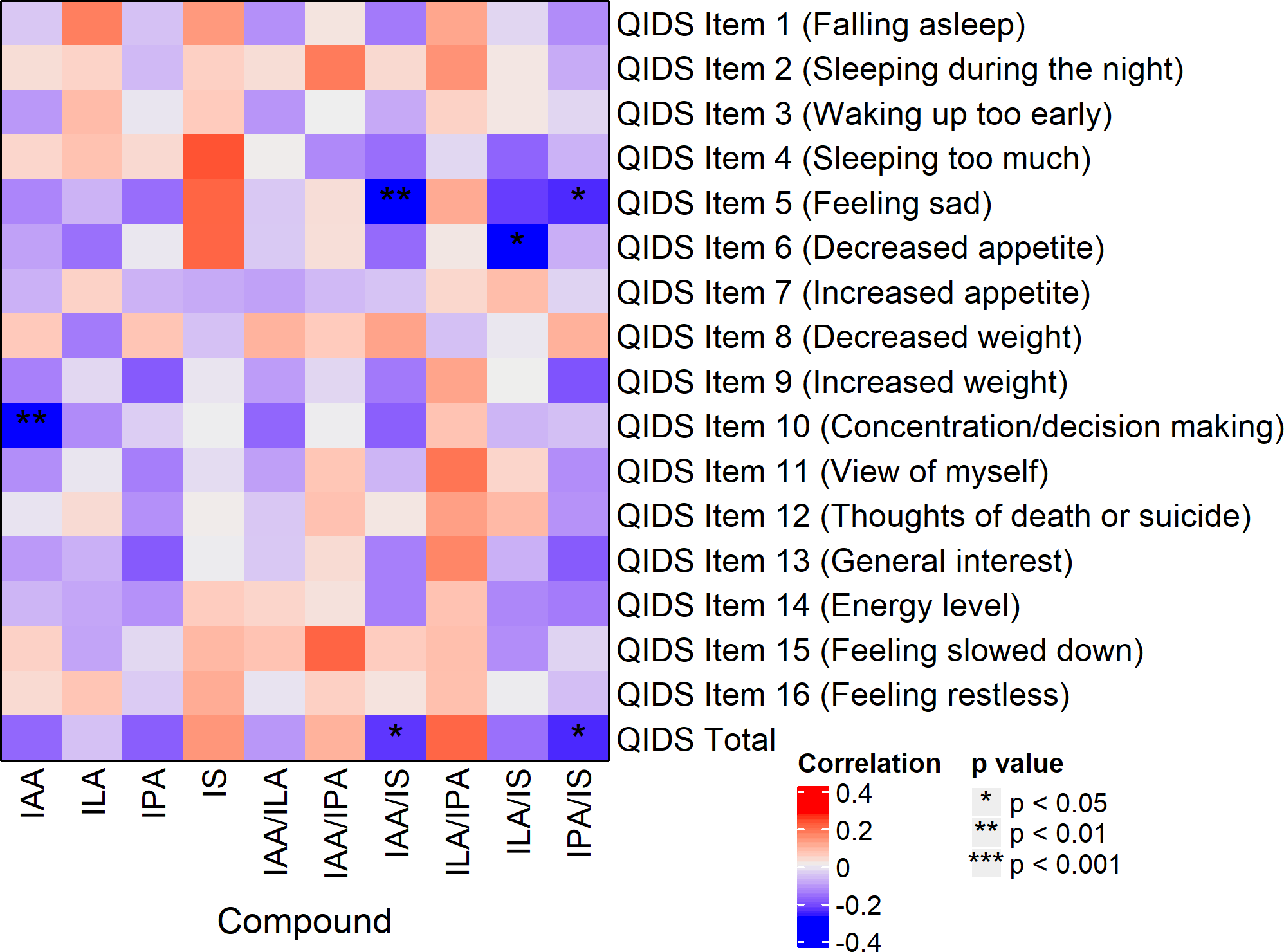

Supplement: Supplementary file 4 — Supplementary Figure 4. [file 41598_2021_99845_MOESM4_ESM.tiff]

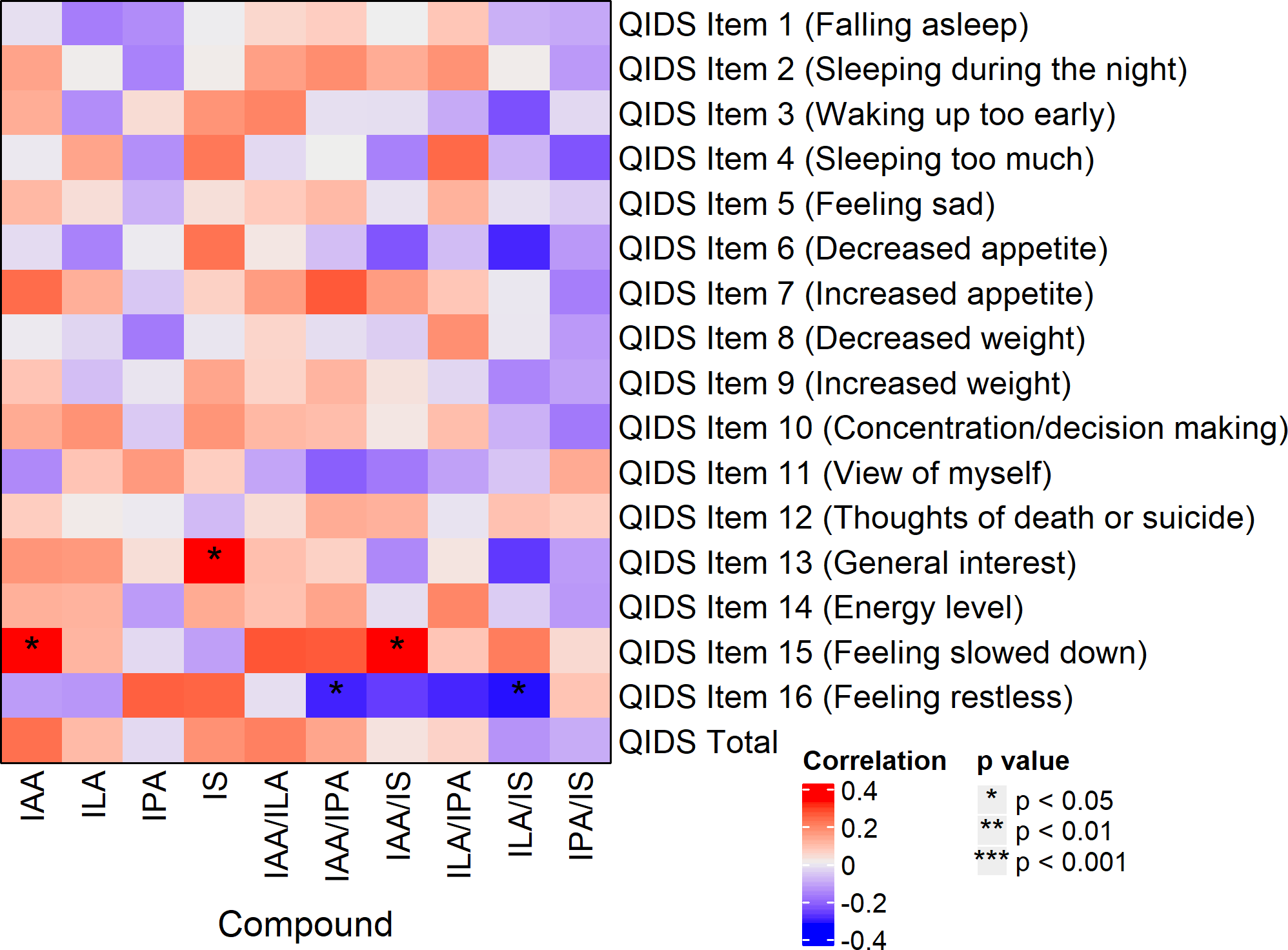

Supplement: Supplementary file 5 — Supplementary Figure 5. [file 41598_2021_99845_MOESM5_ESM.tiff]
