# Supplementary material for: Indoxyl sulfate, a gut microbiome-derived uremic toxin, is associated with psychic anxiety and its functional magnetic resonance imaging-based neurologic signature
Source: Sci Rep. 2021 Oct 25;11:21011. doi: 10.1038/s41598-021-99845-1 (PMC8546034; doi:10.1038/s41598-021-99845-1)
Supplement: Supplementary file 6 — Supplementary Legends. [file 41598_2021_99845_MOESM6_ESM.docx]

**Supplementary Figure 1.** Heat map of Holm-corrected partial Spearman rank correlations between baseline indole abundance/ratio and demographic variables.

**Supplementary Figure 2.** Heat map of Holm-corrected partial Spearman rank correlations between baseline indole abundance/ratio and Hamilton Anxiety scores and Hamilton Depression scores in the female group, after accounting for age and BMI.

**Supplementary Figure 3.** Heat map of Holm-corrected partial Spearman rank correlations between baseline indole abundance/ratio and Hamilton Anxiety scores and Hamilton Depression scores in the male group, after accounting for age and BMI.

**Supplementary Figure 4**. Heat map of Holm-corrected partial Spearman rank correlations between baseline indole abundance/ratio and QIDS-SR items and total score in the female group, after accounting for age and BMI.

*Abbreviations*: QIDS-SR: 16-item Quick Inventory of Depressive Symptomatology – Self-Rated.

**Supplementary Figure 5**. Heat map of Holm-corrected partial Spearman rank correlations between baseline indole abundance/ratio and QIDS-SR items and total score in the male group, after accounting for age and BMI.

*Abbreviations*: QIDS-SR: 16-item Quick Inventory of Depressive Symptomatology – Self-Rated.
